# Experiencing illness as a crisis by the caregivers of individuals with Prader-Willi Syndrome

**DOI:** 10.1371/journal.pone.0273295

**Published:** 2022-09-01

**Authors:** Katarzyna Kowal, Michał Skrzypek, Janusz Kocki

**Affiliations:** 1 Chair of Health Science and Physiotherapy, Jan Dlugosz University in Czestochowa, Czestochowa, Poland; 2 Faculty of Health Sciences, Vincent Pol University in Lublin, Lublin, Poland; 3 Department of Clinical Genetics, Medical University of Lublin, Lublin, Poland; Public Library of Science, UNITED KINGDOM

## Abstract

**Background:**

The behavioural phenotype of Prader-Willi Syndrome (PWS) implies a specific emotional and social-interactive burden for the caregivers of the individuals with PWS. The aim of the study was to perform an in-depth exploratory analysis of experiences of the familial caregivers of individuals with PWS.

**Method:**

The study was carried out using a sociological methodology of the grounded theory (qualitative research). A purposively selected sample of 20 familial caregivers of children/adults with PWS was invited to take part in individual, semi-structured in-depth interviews which included questions pertaining to coping with problems arising from the condition, including its impact on social interactions, as well as to the meanings of PWS.

**Results:**

The core category emerging from our analysis emphasized “experiencing PWS as a crisis”. The phases in the process of experiencing PWS were specified, each of which is characterised by specific cognitive, emotional and social problems, implying relevant requirements in the care of individuals with PWS. I. Crisis in response to the diagnosis; II. Crisis in response to lack of control over the hunger of individuals with PWS; III. Crisis in response to the social milieu’s failure to understand the nature of the condition; IV. Crisis in response to attempts to plan the future of individuals with PWS. The specificity of the PWS caregiver’s experience is primarily determined by the need to reconstruct the entire family’s lifestyle. The experiences of caregivers of PWS persons, at the time when they were available for study, had the characteristics of crisis. Moreover the psychosocial consequences of PWS were not subject to normalization and attempts to attribute any meaningful existential sense to the PWS were ineffective in the time period under scrutiny.

**Conclusions:**

Identifying phases of the PWS experience process from the perspective of the caregivers of individuals with PWS may be used to profile interventions supporting PWS individuals’ families in a manner corresponding to the flow of the illness experience.

## Introduction

Due to their rarity, orphan diseases imply an intensified emotional burden on the caregivers resulting from, among others, uncertainty about the clinical course of the illness, the behavioural phenotypes characteristic of the given disease, or the lack of causative treatment [1–3]. This situation results in deterioration of the quality of life of the patients’ caregivers, albeit the available pool of more specific data pertaining to this topic is highly limited due to, among others, the considerable difficulties in recruiting sufficient study groups and therefore small research samples, which hinders generalization of the obtained results, as well as the lack of standardized, dedicated research tools [4].

In the present study, based on the theoretical premises of sociological symbolic interactionism and using grounded theory methodology [5, 6], an attempt was made to explore the ways in which the PWS is experienced by the familial caregivers of individuals with Prader-Willi Syndrome (PWS). The research focus was dictated by the assumption that people create the meanings of their circumstances by engaging in specific actions. Studies on the illness experience undertaken from such a theoretical perspective must therefore account for actions inspired by the illness and its course in the context of everyday life [5, 7, 8]. Both symbolic interactionism and grounded theory focus on the processual, emergent nature of the analysed phenomena, including those associated with the concept of illness experience. Hence, particular efforts were made in the course of the study to capture the processual character of the analysed phenomena.

Important inspiration for the study was provided by the theses of Timmermans and Haas [9], who suggested reorienting sociomedical research pertaining to the illness experience in such a way as to more comprehensively account for the biomedical factors of phenomena studied by sociologists. This makes it possible to describe not only “*how social processes affect the severity or course of diseases*”, but also “*how*, *in turn*, *specific stages of disease affect social relationships*, *work*, *neighbourhood*, *or family life”* [9, p. 661]. A research approach that accounts for the biomedical characteristics of the disease and its course allows one to explore “*the pathways*, *processes*, *and mechanisms of the dynamic interplay between biological health and social life*” [9, p. 661]. It therefore provides a very interesting cognitive perspective, while also enabling one to generate output with a distinctive applicational potential.

Comment is required on the interpretative framework used in the research and its specific terminology. While making a medical description of PWS, the authors used the biomedical interpretative framework, within which the PWS is positioned as a disease (see the Orphanet database of rare diseases, where PWS is located at item 739, and also the ICD-11 classification, where PWS is denoted as LD90.3). In turn, when discussing the results of our own studies concerning the experiences of caregivers of PWS persons, we adopt and use the PWS-related discourse, which was decidedly dominant in the narratives of the studied persons: all the subjects used the term “illness” to refer to PWS, which shows that the discourse on PWS in Poland, in the studied section of the circle of caregivers of PWS persons, is of biomedical character. It should be emphasized that analogous, biomedical terminology in reference to PWS is also used by the only Polish patients’ organization acting for persons with PWS [Polish Association of Prader-Willi Syndrome]. Therefore, taking into account the specificity of discourse on PWS in the social environment of persons with this condition, the presented research employed the sociomedical interpretative perspective of “illness as an experience”/”illness experience”, related to the interpretive approach in medical sociology, and, as part of the adopted approach, we treat PWS as illness in accordance with the specificity of discourse on it.

PWS is an orphan disease which is the most common genetic cause of life-threatening obesity, whose incidence has been estimated at 1:10.000–1:25.0000 live births [10]. 70%-75% cases of PWS are caused by paternal chromosome 15q11-q13 deletion (genetic type I), 20–30% of cases by maternal uniparental disomy of chromosome 15 (mUPD; genetic type II), while in the remaining 2–5% of cases, the patients are characterized by diverse genetic disorders occurring in the 15q11-q13 region (imprinting defects, ID) [11–14]. The direct genetic cause of PWS is, however, the lack of active paternal genes located on chromosome 15 in the 15q11-q13 region [2].

The clinical course of PWS includes two distinct, characteristic stages: the stage of neonatal and early infant development when the child is diagnosed with varying degrees of central hypotonia, hypogenitalism, sucking reflex disorders, and feeding difficulties resulting in poor weight gain (“failure to thrive”, FTT) (stage 1). The second stage entails the emergence of, among others, retardation of psychomotor development, a low level of physical activity, as well as behavioural feeding-related disorders with hyperphagic features, associated with the occurrence of early onset obesity (obesity is revealed on average at the age of 2) (stage 2) [11, 13, 15]. The transition from stage one to stage two, i.e. the “switch” from feeding difficulties to hyperphagia, takes place between 18 and 36 months of age [acc. to 11].

Miller et al. posit that five nutritional phases ought to be distinguished in the natural course of PWS. They indicate that developmental disorders first emerge in utero (phase 0); next the infant is flaccid rather than obese, and reveals feeding difficulties (phase 1); in the next phase of the natural course of PWS an increase in body mass is observed (phase 2); this is followed by hyperphagia, food-seeking behaviour and lack of satiety (phase 3); in some patients a final stage of the feeling of satiety may also be observed (phase 4) [11].

PWS patients typically fall within a certain specific morphological phenotype, including short stature, small hands and feet, light complexion and hair colour, and sometimes show signs of albinism, as well as underdevelopment of the genitals and a characteristic facial morphology [15, 16]. Approximately 40% of individuals with PWS reveal a mild intellectual disability and around 20% have a moderate ID, however the intensity of intellectual disability depends on the genetic type. E. Roof et al. [17] showed that the subjects with mUPD (genetic type II) reveal higher verbal IQ scores than those with deletion (genetic type I): only 17% of subjects with type I had a verbal IQ ⩾ 70, while 50% of those with mUPD had a verbal IQ ⩾ 70. The behavioural phenotype of PWS may furthermore include obsessive-compulsive disorders, self-injurious behaviour, and depression [13]. Certain phenotypical differences can be observed between genetic type I and type II patients, specifically, genetic type I is characterized by intensification of the behavioural characteristics typical of PWS (focus on food, mental retardation), whereas genetic type II entails better cognitive functioning of the PWS children and less intensive behavioural problems; however, the later type is also characterized by the incidence of additional disorders in terms of visual acuity and stereotypic vision, which are not experienced by type I individuals [13].

Furthermore, the typology and intensity of psychopathologies varies depending on the genetic type of PWS. Affective psychoses occur more commonly in the cases of mUPD as compared to the cases of PWS associated with deletion (60% vs. 20%), whereas depression is equally common in both genetic types [18]. Dykens et al. did not observe behavioural differences between individuals with the respective types of deletion (type I vs type II) [12], while in a study by Butler et al. including PWS patients with both types of deletion and mUPD, a greater intensity of behavioural and psychological problems was reported in persons with type I deletion as compared to the other genetic types of PWS [19].

The primary feature of the PWS behavioural phenotype is an insatiable, voracious appetite which, combined with a gag reflex disorder and in the absence of the caregivers’ efforts aimed at controlling the diet, may lead to treatment-resistant obesity [3, 15, 16]. In a study by Lindgren et al. [20] it was demonstrated that compared to persons with both normal body mass and obesity, PWS patients manifest eating patterns characterized by non-decelerating eating curves, which testifies to a reduced sense of satiety [20]. The results of the mentioned study suggest that abnormality of the satiety response may be responsible for the hyperphagia in PWS. Furthermore, PWS individuals typically show a distorted correlation between the supply of calories in their meals and body mass, consisting in the fact that fewer calories are required to maintain a constant body mass, while weight reduction necessitates greater caloric reduction when compared to simple obesity [15]. It has been pointed out that the low energy expenditure in persons with PWS is due, among other factors, to their relatively low fat-free mass content [21]. Compulsory eating in the course of PWS may be directly life-threatening due to stomach rupture [15].

The behavioural eating problems constitute the primary, life-long factor of the behavioural phenotype of PWS individuals, requiring food-related supervision [14]. The insatiability of appetite is accompanied, in most individuals, by an increased incidence of temper tantrums and rages [15]. The everyday life of families tends to be focused on the struggle to effectively control the PWS person’s eating habits; where these efforts fail, a child’s body mass can reach up to 113–126 kg by their late teens [15]. Butler and Thompson show that approximately 1/3 of all PWS individuals weigh more than 200% of their ideal body weight [13]. As a consequence, the lives of persons with PWS and their families are “*trapped in a food-oriented world*” defined by the constant struggle to limit the food intake [15].

A review of the literature pertaining to the experience of the caregivers of persons with PWS reveals a relatively sparse pool of available data. The unique character of the caregivers’ burden is determined by the specific constellation of behavioural problems and psychopathologies, comprise the behavioural phenotype of PWS [16]. While the literature on the subject describes the stages of the natural progression of PWS, little is known about how the illness experience of their caregivers’ changes during the course of the condition. Analyses have been conducted with regard to, among others, psychosocial problems, coping strategies and the demand for information among parents of PWS children in comparison to parents of children with Angelman syndrome [22]. The correlates of parental stress were analysed for the parents of children with PWS and other genetic syndromes [23], problems related to stress and social support available to parents of PWS children [24] as well as the quality of life of families with children with PWS and the impact of the condition on healthy siblings [25].

In the context of our own research, the results reported by Mazaheri et al. [25] are particularly important as they document the impact of PWS on the entire family system. The qualitative part of the study documented the diversity of the distress experienced by mothers caring for children with PWS and the complexity of the PWS patient’s needs as source of chronic stress. The study emphasized, among others, the burden resulting from the need to ensure continuous treatment of the child with PWS (growth hormone injections, insulin administration, etc.), to constantly monitor the child’s eating habits (this ordinary daily activity in the case of PWS is a direct threat to the child’s health and life), as well as to cope with behavioural disorders and psychopathologies. A significant factor contributing to the caregivers’ emotional strain and sense of powerlessness stems from the fact that PWS is incurable [25]. Studies reveal that looking after a PWS person is more difficult and entails a greater burden than tending to children/persons with other health problems, including mental disabilities [24], Fragile-X or Williams syndrome [3, 26]. In a study by Kayadjanian et al. [27], it was reported that a particularly high level of caregiver burden is associated with the care of teenagers and young adults with PWS, while lower levels are attributed to tending to older adults over the age of 30 and the youngest patients. Providing care to a PWS person disrupts relationships between spouses, the ability to work, or sleep, and induces depression [27]. The available literature on the subject includes certain studies that draw attention to the perspective of the caregivers of children with orphan diseases, but without specifying the specific criteria for inclusion in the research. For example, in the studies of Anderson et al., the leading problems associated with looking after a child with an orphan disease were identified [28]. The dominant methodological approach in this area of research tends to entail conducting a quantitative survey study. The main issue with this method stems from the fact that its scope, by definition, is limited only to those problems that are included in the questionnaire. This means that it risks omitting certain issues which may be significant to the families but are not covered by the survey.

Given the fact that constructing effective systems of support in rare diseases necessitates taking into account the perspectives of both the affected individuals themselves and their caregivers [29], as well as in response to the highly unique perspective and experience of the caregivers of persons with PWS, emphasized in the guidelines for PWS-related research formulated during the 2015 PWS Mental Health Research Strategy Workshop [18], a qualitative study was undertaken to analyse the experiences of caregivers looking after individuals with PWS. The starting point for the discussed study was the premise that given the staged nature of the condition’s natural course [11, 13, 15], the families of persons with PWS experience a crisis of a processual and long-term nature. It was assumed that the experience of PWS patients’ caregivers is characterized by a specific cognitive perspective, namely one where the state of feeling “relative security and peace” is questioned and replaced by a state characterised by a sense of threat to the existing balance [30, p. 17].

## Material and method

### 1. Study design

The presented study was conducted as part of the research project entitled “The experience of Prader-Willi Syndrome from the perspective of caregivers of an ill person–a sociomedical study of a rare genetic disorder”. The aim of the study was to describe the PWS experience from the perspective of the caregivers of affected persons, specifically to: discover the ways in which the respondents perceive various PWS-related aspects of their everyday lives, determine how PWS impacts the functioning of families, identify the potential problems implied by PWS in the context of intra-family communication, specify problems related to the identity of caregivers of individuals with PWS, as well as characterize social functioning of a family whose member experiences PWS.

The primary research problem was expressed in the following question: How is PWS experienced by the caregivers of an affected child/adult in the non-medical context of everyday life? The thus formulated research problem was approached by means of a qualitative sociological study based on the grounded theory method in the constructivist perspective proposed by Charmaz [31].

### 2. Participants

Selection of the respondents was intentional and was determined by the criterion of being a guardian of a patient medically diagnosed with PWS. The research group was composed of 20 caregivers of PWS children/adults, of whom 17 respondents were women (85%) and 3 (15%) were men (Table 1). At the time of the study, the caregivers were aged between 30 and 56 years, mean 41.95, SD = 6.85. The relevant PWS patients were aged, at the time of the study, between 4 and 26 years, mean 13.80, SD = 6.83, with 6 (30%) females and 14 (70%) males. Overall, the group of PWS patients was composed of 13 persons under the age of 18 and 7 adults. In all the individuals the PWS diagnosis had been genetically confirmed; they all lived at homes with their families. The average age at diagnosis was 17.95 months (range 1–60 months), SD = 18.23. The distribution of individuals with PWS in terms of the genetic type of the illness was as follows: 14 persons (70%) with de novo paternal deletion of the chromosomal region 15q11-q13 (genetic type I), including 4 females and 10 males; 6 persons (30%) with UPD (genetic type II) including 2 females and 4 males; in the group of individuals with PWS 80% had a diagnosis of imprinting defects (ID).

**Table 1:** Characteristics of research group of caregivers and individuals with PWS.

**Caregivers’ characteristics**	
**Gender**	
Male	3 (15%)
Female	17 (85%)
**Age**	from 30 to 56 mean 41.95 SD = 6.85
**Characteristics of individuals with PWS**	
**Gender**	
Male	14 (70%)
Female	6 (30%)
**Age**	from 4 to 26 mean 13.80 SD = 6.83
**Age at diagnosis** *(months)*	from 1 to 60 mean 17.95 SD = 18.23
**Genetic type I**	14 (70%)
**Genetic type II**	6 (30%)
**ID**	16 (80%)
**BMI of individuals below 18 years old**	from 12.77 to 23.46 mean 16.52 SD = 3.03
**BMI of adult individuals**	from 19.49 to 46.88 mean 28.44 SD = 9.77

The nutritional status of the PWS patients was assessed by calculating their body mass index (BMI) [kg/m^2^], which in the case of persons under the age of 18 was analysed relative to the current Polish developmental norms for BMI [32], and in the case of adults, to the WHO classification. Among the individuals with PWS under the age of 18 (n = 13), in terms of Polish standards, nine patients had normal body mass (69.23%), three children were underweight (23.08%), and only one was overweight (7.69%). Of the seven adults with PWS, four had normal body mass, two were classified as class 1 obesity and one as class 3 obesity, as per WHO classification.

None of the subjects had any relationship with the researcher, they had no contact with each other before the study commenced. Moreover, the researcher had neither personal experience nor personal reasons for involvement in a research project on this subject. This information was also presented to the persons invited to partake in the study. The researcher’s interests in the subject of the project were only substantive.

### 3. Data collection

The study was conducted between July 1 and September 30, 2019. Interviews with the caregivers of people with PWS took place outside the medical cent res where the PWS individuals were being treated. The interviews were carried out by telephone. Based on pilot studies carried out by means of personal meetings and telephone conversations, it was established that interviews conducted by telephone/telephony application rather than face-to-face meeting gives the respondents greater psychological comfort and thus the possibility of deeper penetration into the reported experience by the researcher. The consent of each respondent was obtained prior to their inclusion in the study, who was asked to indicate the optimal date and time of the conversation for them so that they were in a quiet place, without the presence of non-participants of the study (including first and foremost persons with PWS). None of the persons invited to participate in the study refused, nor resigned during the course of the interview. An individual in-depth interview was conducted with each of the caregivers, in accordance with Charmaz’s definition of an intensive interview which as a qualitative research method with high interpretative potential, allows in-depth examination of the relevant problem [31: p. 25–35]. In order to obtain detailed and targeted empirical data that comprehensively illustrate the analysed issue, the respondents were encouraged to not only describe experiences related to caring for individuals with PWS with respect to the topics suggested by the researchers, but also to provide their own insights and interpretations of the events related to the condition and its course. The interviews followed a previously prepared general script, which included general, open questions concerning the PWS experience as viewed from the caregiver’s perspective. The research tool (the list of informative needs) was based on the results of the pilot phase of our research. Its core element was the interview with the mother of a child with PWS, who later served as a “doorman/doorkeeper” in this research as she provided the researchers with the contacts with the representatives of the PWS community in Poland. We applied in the research tool the wording from this interview including naming PWS as illness. Once the respondent opened up to the thus constructed questions, the researchers proceeded to explore more specific themes by referring to a set of supplementary, partially structured questions. With the view of obtaining the most comprehensive, precise and true information possible, the researchers asked detailed questions focusing on the given discussed thematic point, which concerned experiences related to the events and situations described by the respondent, as well as the significance attributed to them. In the course of the conversations, the respondents were continuously encouraged to share their experiences related to the discussed topics, including those personal or even intimate in nature; efforts were made to ensure optimum conditions and venues for this purpose. The respondent’s answers were registered using a voice recorder, to which each respondent agreed. During the interview, the respondents usually remained in their own homes. The duration of the interview ranged between 60–90 minutes. The empirical material obtained in the study was subjected to naturalized transcription (a copy of spoken discourse). Due to the fact that the collected research material proved to be extremely rich in data, there was no need for repeated interviews. Attached to the manuscript, a list of the researcher’s information needs was presented, which was the tool for conducting the in-depth interviews with the caregivers of individuals with PWS (S1 File).

The interviewer was the first author of this text, a sociologist by profession. Katarzyna Kowal is a doctor of humanities in the field of sociology with specializations in: 1) sociology of health, illness and medicine; 2) body sociology; 3) qualitative sociology. For 20 years she has been conducting sociological research embedded in the interactional and interpretative paradigm.

The study was conducted under the consent of the Bioethical Committee of the Medical University of Lublin—resolution No. KE-0254/253/2019 (S2 File).

### 4. Data analysis

The analysis of the collected qualitative data was carried out in accordance with the principles of the grounded theory methodology by Kathy Charmaz [31], which determined the following stages of the conceptual work: 1. initial "line-by-line" coding in the form of verb coding; 2. concentrated coding to select the most analytically significant codes, which also took the form of verb coding; 3. theoretical coding ensuring integration of the codes generated in the concentrated coding stage and allowing entry to the conceptual category level; at this stage of coding, the researcher reached for the code family *Process* by Barney G. Glasser [33: p. 74–75]. One coder participated in coding, which was the first author of this work [K.K.]. During coding, which principally entailed defining the given data content [31: p. 186–187], the researchers remained open to new potential themes that could emerge in the course of data processing. This attitude was consistent with procedures stipulated by grounded theory methodology with regard to preserving the context of discovery (serendipity). In principle, this approach facilitates the discovery of phenomena which the researcher was not initially searching for [34: p. 27]. After completing the coding activities, the researcher switched to writing notes as the main method of the grounded theory, which is extremely useful for explaining and filling in the generated theoretical categories of the already emerging theory.

In the discussed analysis, the research category of the effects of the PWS became the central concept. It was found that from the perspective of the caregivers of persons with PWS, the effects take the form of a long-term crisis. For this reason, the analysis of qualitative data was focused on describing the process of experiencing crisis by the caregivers of PWS individuals. In the next stage, the conceptual analysis was elevated to a more abstract and theoretical level; ways in which crisis is interpreted by the caregivers were identified, its phases were described, taking into account the significance attributed to the individual phases of the PWS experience. In line with the adopted methodological premises, the interpretative perspective of caregivers of individuals with PWS was accepted as the leading one. The researcher’s activity was not linear, i.e. the activities of data collection and analysis were mutually overlapping. Theoretical sampling, which requires a turn towards the empirical world, was the strategy employed until theoretical saturation was reached. This was synonymous with the fact that subsequent interviews no longer provided any new data within the framework of the generated theoretical categories [31: p. 96–115]. The credibility of the results below is confirmed by feedback from research participants, whose meanings and perspective coincide with the interpretations of the authors of the text. We obtained them thanks to the use of the theoretical sampling procedure in the initial and final phase of the research, together with the method of continuous comparison [31: p. 96–122], which assured us of the significance of the generated theoretical categories and made it possible to extend their scope. We also became convinced that the crisis category should function as the main concept in the analyses. We received additional feedback from the participants in the study, which strengthened and clarified the already focused category of the crisis experienced by the caregivers of individuals with PWS, after sending them the preliminary version of the research report.

## Results

Analysis of the obtained empirical material revealed certain fundamental similarities in the accounts of the caregivers of individuals with PWS, which seems to corroborate the viability of a crisis experience in the context of these families. The cited research results, representing classic accomplishments in the sociology of illness experience, constituted the theoretical framework for our own studies; however, due to the requirements of grounded theory, they do not serve as a preconceptual theory. The authors did not aim to verify the theories of Davis, Strauss, or other researchers but rather engaged in a parallel discourse, while merely referring to certain terms and concepts introduced by the same. Similar to the experience of parents of children with poliomyelitis, as discussed in the classic study by Davis, our research allowed us to identify phases of the PWS experience, related to the ongoing emergence of the PWS behavioural phenotype. In Davis’s study, the starting point was to define crisis as “*a relatively sudden and unanticipated disruption*, *of extensive and protracted significance*, *in the everyday activities*, *understandings*, *and expectations of a social unit*, *in this instance the family”* [30, p. 17]. In his analyses Davis posited, given the similarities of the experiences of the examined families, that one should focus on the “*essential or model crisis experience*” [30] and proposed an approach within which he distinguished the respective stages of the crisis experience (“*a sequential-stage analysis*”). This approach enabled analytical ordering of the empirical data [30]. Similarly, the experiences of the families discussed herein are temporal in character and follow a certain specific trajectory. The term draws upon the observations of Strauss et al. and their concept of illness trajectory. To quote the original publication:

*The term trajectory focuses us on the active role that people play in shaping the course of an illness*. *This course is shaped not only by the nature of an illness and a person’s unique response to it but also through actions taken by health personnel as well as the ill*, *their wives or husbands*, *and any others involved in its management* [8].

The proposal of Strauss et al. [35] allows a simultaneous approach to the course of an illness from the biomedical perspective as well as the perspective of active efforts undertaken by the individual and his/her social milieu with the aim of controlling or shaping the said course. Both of these aspects of a chronic illness are depicted through the prism of the two primary concepts introduced by Strauss et al., namely the “course of illness” and “trajectory of illness”. The analytical approach proposed by Strauss at al. allows one to capture the specificity of the activities undertaken by the families of PWS patients, whose main goal is to modify the natural course of the illness in such a way so as to prevent morbid obesity and death of the patient due to obesity-induced complications. The interactionist analytical category of “work” was therefore deemed as viable in analysing the experiences of caregivers of individuals with PWS. In the original approach of Strauss et al., the term “work” was used to refer to efforts aimed at shaping the course of the illness,

*[…] designed to manage one or more aspects of the illness and the lives of ill people […]* [8].

Analysis of the collected empirical material allowed us to identify phases in the illness experience of the caregivers of individuals with PWS.

### I. Crisis in response to the diagnosis

The moment of the diagnosis, specifically on learning about the genetic testing results, the process of experiencing PWS for the caregivers begins. A characteristic feature of this phase of the crisis is confrontation with emotions accompanying the diagnosis of an untreatable disease. The caregivers go through shock and disbelief, and the intensity of the experience tends to be greater the later the diagnosis is made. Here are some examples of what the interviewed caregivers said:

*I was in shock*! *I cried*. *I started calling the doctor names*, *telling him he was crazy*, *that he was making up my child’s illness*. *5 years after the child was born*, *he comes up with some genetic disorder*. *Until then*, *the neurologist had told us our child was developing well*.

(Interview 8)

*It was a shock*, *I remember I couldn’t stop myself from crying*. *We lived in [*…*]* (here the caregiver gives the name of the city—note by K.K.) *because we were treated there*, *so I went home with the diagnosis*. *I couldn’t wait to come home and finally cry*, *to let it out*. *My son was 3*.*5 years old at the time*, *so it was even more difficult to accept*. *I knew then that there was no treatment*, *that it was a illness for the rest of his life*.

(Interview 12)

*Shock*, *for sure it was a shock at the beginning*, *questions like why us*, *why did it happen to us*? *Already when the genetic tests confirmed that it’s Prader-Willi*, *there was shock*, *disbelief and anger*. *We didn’t immediately accept this diagnosis*. *Only after a year*.

(Interview 13)

The strongest experiences accompanied the caregivers over the initial several months after the diagnosis. In the accounts of the respondents, the predominant feeling at this stage of the illness experience is one of fear for the child’s wellbeing. Their everyday lives are shaken by the PWS diagnosis, anxiety and uncertainty continue to mount. When the child was diagnosed with the rare genetic condition, the parents found themselves completely unable to act for a time. Their first reaction, after overcoming that specific behavioural numbness, was a desperate search for other parents whose children had also been diagnosed with PWS. The examined respondents expected to gain through these contacts solace, reassurance and support in coping with the strongly experienced negative emotions, but instead, those contacts only exacerbated their experienced crisis:

*I was terrified after the first contact with a mother whose son was still under aged but already so overweight that he’d already had a collapse*. *(…) Nobody tried to tell me I would do fine*, *quite the opposite in fact*, *they’d say I needed to be patient because the worst was still ahead of me*. *Nobody patted me on the back or tried to comfort me*, *from the very beginning*, *I was told it was going to be hard*. *Someone told me for instance I would have to put a padlock on the fridge*. *(*…*) Then I went to my first convention of the society to help individuals with Prader-Willi Syndrome and it was there that I understood what awaited me*. *I don’t remember much about that convention because I cried throughout the whole meeting*. *It just dawned on me that something might be ahead of us*, *some other life*. *This aggravated my crisis*. *It was a shock for me to talk to other parents*, *to actually come into contact with people who have it*. *It was*… *it was the biggest shock*.

(Interview 10)

*At the beginning of my son’s illness*, *right after we found out*, *I met a "prader" who was twenty years old*. *I was shocked when I saw him*, *my jaw dropped*. *He was very obese and had a deep intellectual disability*. *His caregivers told me that they were going to put him in a shelter*. *They didn’t treat him like a normal human being*, *they called him names*, *poked him*, *just a tragedy*. *I was terrified by this illness and I was afraid that I might treat my son like that too*. *(*…*) I even told my wife after that that we might put him in a center*.

(Interview 19)

During this phase, the caregivers’ sense of crisis is further exacerbated by concerns stemming from the immediate need to start coping with the illness. Particularly the necessity to diametrically alter the lifestyle of all the family members and the inevitable changes to the very foundations of family life lead to mounting anxiety and insecurity. At this phase of the crisis, the parents tend to ask existential questions, e.g.: “why has this happened to my child?”. As the thus formulated question cannot be viably answered within the framework of lay interpretative systems, the caregivers experience anger and a sense of helplessness. The emotional response is followed by an attempt to understand the medical aetiology of PWS and process that medical knowledge in the context of one’s own, individual situation. The respondents showed a very strong desire to understand the causes of the genetic disorder diagnosed in their own child. This need turned out to be unsatisfied in many cases, mainly because the respondents firmly rejected the explanations offered by medical professionals, or they did not receive these explanations at all:

*I started asking myself this question*, *why me*? *Why my son*? *But for several years I couldn’t answer it*. *It definitely bothered me*. *The geneticist told us that our son would not go to kindergarten*, *he would not go to school*, *he would not achieve anything in life*, *he would want to eat all the time and it would be best to put him in a center*. *This was the information I received*, *it was very depressing*. *And that’s it*. *Nothing about the causes of this illness*. *I didn’t accept it*. *I searched encyclopedias and the Internet*, *but then there was very little information*. *I wanted to understand why this rare illness happened to us*.

(Interview 1)

*The geneticist told us it was a bit like winning the lottery*. *But I still have no idea why this happened to us*??? *It keeps running through my head*, *why me*, *why did it happen to me and my child*??? *Why are other people’s children healthy*? *I really hate it when people say it’s a disease that only happens to good parents*. *I always reply that if so*, *I wish I’d killed someone or stolen something*, *then I’d be bad and maybe my child would not be ill*. *That’s a ridiculous comparison*. *If you’re a good person your child will be sick*, *and if you’re bad your child will be healthy*. *I know there must be other reasons; I don’t believe in this kind of coincidences*. *Something must have been wrong with me*, *or my husband*.

(Interview 9)

*I asked myself*, *why me*? *Why has this happened to my child*, *my family*? *Questions unanswered*! *We know everything from the medical point of view*. *I have read everything about it*. *Because genetics say it’s completely unpredictable*, *the father’s genes must hit the mother’s susceptible genes*, *so it’s incalculable*, *unpredictable and that’s it*. *We had no influence on it at all*. *Nothing*! *Didn’t matter what we did*. *The doctor said so*. *It’s genetics*, *but I was looking for a deeper cause*, *a cause that would have had an effect even earlier*.

(Interview 10)

Accepting the professional aetiology of PWS was difficult for the respondents also because medical interpretations did not attempt to attribute responsibility, did not point to the person whose “fault” it is that the child has genetic disorders. The respondents’ interpretative activity revolved mainly around questioning the interpretation offered by medical practitioners who explained that the child’s illness was simply a matter of chance, bad luck, a sort of “genetic roulette”. Ignoring the medical interpretation of the PWS aetiology, the respondents continued to compulsively search for a causative explanation for their child’s condition. The attempts to place the alleged “responsibility” or “blame” for the condition lead to marital conflicts. Here we are dealing with a situation, where in the process of striving to gain “full understanding” of the PWS causes, the medical interpretations of the illness’s aetiology are ultimately replaced by lay explanations. The equilibrium of family relationships is upset not only by the blame mutually placed on each other by the parents, but also blame placed by the caregivers on themselves:

*There were such situations with my husband that we simply blamed each other for the child’s illness*. *In general*, *we had a grudge against each other for everything*. *But it was simply out of our powerlessness and helplessness*, *There were times when we just gave up*, *we were helpless*. *We asked ourselves what we had done wrong to make this child ill*? *Because this illness took so much from us*.

(Interview 1)

*We went through a stage of blaming each other for our daughter’s condition*. *My husband first claimed it was my fault*, *then proceeded to blame himself*. *By coincidence*, *we were renovating our flat while trying to have a child*, *so we ended up berating ourselves for using all that paint*, *for all the things that went on at our place*, *because of that our daughter was ill*. *One day*, *I would insist I was to blame*, *that it was something wrong with me*, *the next*, *my husband would say the fault was his*.

(Interview 13)

*There were quarrels about it many times*, *especially at the beginning*, *when we had not yet got used to the illness and there were no other children*. *And this is your fault*, *and this is your fault*, *there was such shifting of blame*.

(Interview 19)

The disruption of family relationships also extends to other relatives. Since the most common genetic type of PWS involves the deletion of a paternal chromosome fragment, the diagnosis often results in blame being placed on the child’s father:

*My mother doesn’t understand this illness*, *she silently blames my husband for it*. *Well*, *because*… *(*the respondent mentions the child’s name here—researcher’s note K.K.*) has a deletion*, *that is this paternal mutation*, *so in my mother’s opinion the blame for it lies with my husband*. *My mother doesn’t understand it*, *and my brothers and sisters-in-law don’t understand it either*. *That is why our contacts*, *among other things*, *are limited*.

(Interview 9)

*My in-laws were against me*, *they blamed me for our son’s illness*. *They said it was my fault*. *And so*, *I had to live with that guilt*. *For years*, *my mother-in-law would not as much as say “good morning” to me*. *It was hard*.

(Interview 19)

Notably, the tensions and conflicts continue to upset family relationships as long as the commonsensical interpretations of the disease’s aetiology remain predominant. Interestingly, the analyses of the collected empirical material do not reveal that analogous situations occurred in the case of children affected by maternal disomy, i.e. genetic type II of PWS.

To recapitulate the abovementioned analysis of this stadium of the crisis experience emerging in response to the medical diagnosis of PWS, it should be noted that most of the aspects discussed above pertain to a model situation where the medical diagnosis is made in the first months of the child’s life. This context does not necessitate an immediate drastic shift in the family’s life and its overall functioning to implement eating restrictions and the entire regime related to properly looking after a PWS child. At this phase, the experience of crisis is dictated primarily by the caregivers’ internal struggle, interpretative work focused on understanding the present and future ramifications for the child and the entire family, as well as anticipation of the expected outcomes of the illness.

### II. Crisis in response to lack of control over the hunger of individuals with PWS

Analysis of the empirical material indicates that the phase of crisis whose main determinant is the reaction to the lack of control over the child’s hunger occurs once the caregivers have managed to accept the medical diagnosis. A sign of the said acceptance, of coming to terms with the situation, comes in the form of more normalized interpretations of the illness as “a lesser evil”, “a blessing in disguise”, because “after all, it could have been much worse.” Given the anxiety preceding the medical diagnosis, many caregivers accepted it with relief as, on the one hand, it somewhat reduced their uncertainty concerning the child’s situation, and on the other, it assured them that the child’s life was not in immediate danger. This is evident in the following statements:

*We were actually glad to hear the diagnosis as the doctors had warned us of much more serious illnesses*, *saying our child might not survive beyond six months*. *We were happy because it meant our child would live*.

(Interview 5)

*There was a temporary shock after hearing the diagnosis*, *but then I was relieved to know what it was*, *that although there’s no treatment for this illness*, *our child will live*. *Until that moment*, *I knew that something was wrong with the baby*, *and I didn’t know what*. *I imagined terrible illnesses*.

(Interview 12)

The phase of crisis resulting from the effects of hyperphagia characteristic of PWS was mentioned by the surveyed parents with respect to two, clearly distinguishable periods: when the child was between 3 and 5 years old or between 13 and 15 years old. Analysis of the empirical data reveals that the onset of the caregivers’ crisis related to powerlessness in the face of the child’s compulsive appetite is strongly conditioned by the child’s degree of intellectual development. In the experience of parents whose children reveal moderate intellectual disability (genetic type I–deletion), the moment tends to occur later (i.e. when the child is 13–15 years of age), whereas in the case of parents whose children experience only a mild disability or remain within the intellectual norm (genetic type II–disomy), this crisis phase tends to occur earlier, sometimes even immediately after the child acquires the ability to eat independently (at the age of 3–5 years). The interviewed caregivers reported that the exchange of information among parents regarding the relationship between the chronology of food-related behavioural disorders and the severity of intellectual disability leads to a paradox, where the parents of children with a more serious intellectual disability feel relieved and are more willing to accept their children’s situation as the food-related behavioural problems tend to occur considerably later. What is equally important, parents’ satisfaction also comes from the fact that this group of children is less able to independently seek access to food products, and less frequently and less sophisticatedly resorts to fraud and theft related to it:

*As a mother of a teenage child*, *with as much experience and knowledge as I have*, *I will only say that I prefer his significant intellectual disability*, *his limitations because at least we didn’t have this hunger from the beginning*, *we could prepare for it*. *I am glad that my son*, *although he is 18*, *listens to me and still walks with me holding my hand*…. (here the name of the child is mentioned—note by researcher K.K.) *is a child who doesn’t speak*, *doesn’t write*, *and doesn’t function independently at all*. *My assumption is that our children should be deliberately limited in some way and a bit stupid*, *an ugly word*, *because the wiser they are*, *the more difficult it is for them and for us too*. *I am happy because my son can’t manage to steal food*, *he is always caught by us*.

(Interview 1)

*In our case*… (here the name of the child is mentioned—note by researcher K.K.) *is developing well intellectually*, *has slight mental retardation and he can use it to obtain food*. *For my son*, *since he was 3 years old*, *life has revolved around food and he doesn’t think or talk about anything else*, *only about food*. *He counts what he’s already eaten*, *wonders what else he can eat*, *what to fill up on*. *He is occupied with food 24 hours a day*. *If he were really only relying on me*, *if he had a more profound handicap*, *it would be different*. *When it comes to getting food*, *he’s got so many different methods and behaviors that would never occur to anyone that you can contrive so much to get food*. *PWS is the most severe genetic illness for the environment and family that probably exists*. *We’re already so tired of this illness*, *our whole family has been in crisis for many years and we have signed up for family therapy*.

(Interview 2)

The child’s development in terms of taking control of their bodies with regard to independently moving around and eating makes parents aware of the extent of control they have to exercise over the ill child. They come to realize that their work on the illness, previously focused on coping with specific symptoms, needs to be updated. The everyday lives of the family must be thoroughly reformulated as the full behavioural scope of the illness starts to manifest itself. The seemingly familiar, “domesticated” world of PWS reveals a new side that once again brings about fear and a new level of uncertainty. At this phase of the illness, formerly unknown aspects of the PWS individual’s behaviour come to light and intensify the crisis experience. The triggers of this new phase of crisis may include behaviours such as the first time the child steals food from a store, becomes ill after overeating or eating food from the garbage, chokes on food, or experiences poisoning after ingesting large amounts of medicines. The occurrence of such new and unexpected situations may cause the parents to question the sense of further work on the illness. They inevitably begin to doubt their ability to cope with the challenges inherent in the responsibilities of caregivers of people with PWS:

*When the police brought my son home after he’d stolen something from a store*, *I experienced a serious crisis*. *I realized I would always have to be a few steps ahead of him*, *to predict everything because if I failed even once the police would be back again*. *I went into this spiral*, *thinking I just couldn’t do it*! *It was then that I realized the terrible truth that my son would never be independent*, *that he would never be capable of it and to that moment*, *as a mother*, *I still really wanted to believe he could*.

(Interview 10)

*At the moment when the thefts of food started in the shops*, *the collecting of cigarette butts from the street started*, *when the police first brought*… (the child’s name is mentioned here—note by researcher K.K.) *after shoplifting*, *then brought him a second time*, *I realized that he can’t go anywhere alone*, *he can’t even go for a walk with the dog alone*. *He will have to be driven everywhere*, *he’s got to be under control all the time*, *a 24-year-old guy*! *I was afraid*, *could I do it*? *Can I do it*? *This 24/7 control forced me to change my life to such an extent that a month ago I closed my business and only took care of my son*. *Also I’m not working anymore*! *PWS overcame my professional career*.

(Interview 12)

*When we didn’t have a locked kitchen yet*, *our son took pancakes out of the trash*, *it was the first time that he had eaten something from the bin*. *Then something even worse happened because he ate a whole packet of pills*, *it was vitamins for an adult*, *there were 100 pills there*, *and he was a small child*. *We ended up in the hospital*. *Then he forcibly stuffed himself with food and choked*. *Hospital again*. *There have been several such situations*. *The question is what to do next*? *I was very scared then because I felt that I was losing control over it*.

(Interview 1)

A typical aspect of this phase of the PWS experience is the caregivers’ growing uncertainty as to whether they will be able to maintain full and absolute control over the PWS individual and his/her eating habits in situations where the ill person starts to be cunning and uses new and more effective strategies in gaining access to food. The analysis reveals that the parents’ experience is marked by a mounting sense of helplessness when faced with the PWS individual’s growing disregard for previously established and so far effective dietary regimes:

*My son’s diet had to be limited to 1*,*000 calories*, *but he was a small child then so we restricted his food*. *But he was clever from a young age*, *fishing food out of the garbage*, *eating dog food*. *In time*, *we realized that we would have to lock the pantry*, *put a padlock on the fridge*, *everything had to be locked so he would not get in*. *Then he discovered money and we needed to hide our wallets*, *otherwise he would get up at night and steal money*. *He still does in fact*. *We all have to keep our wallets hidden at night*. *He was very eager to take the dog out for a walk*. *And when he was out there*, *he first started going through the dumpster to look for food there*. *Then he had a stage when he would stand in front of a store and beg for money to buy food*. *Finally*, *during one of the walks he sold the dog*, *went to the supermarket and bought himself some sausage and bread*. *The police went looking for him then and we got charged with neglect*. *At that point we realized that we were losing control of the situation*, *we were being defeated by our own son*. *A child with Prader-Willi syndrome is the perfect thief*.

(Interview 19)

*The most difficult symptom of the illness for me*, *as a mother*, *is the searching for food*, *when my daughter starts it no arguments work*. *It becomes impossible to take away anything she lays her hands on as she reacts with aggression*. *She will throw chairs at me*, *swear*, *hit me in the face*, *pull my hair*. *Taking food away from her is simply impossible and I feel completely helpless then*, *so I have to do what I can to prevent such situations and not allow any food to come within her reach*. *And the crisis comes from my complete physical and mental exhaustion*. *I sleep with the key to our flat under my pillow*, *and she still manages to steal it from me at night and go begging for food in the neighbourhood*.

(Interview 17)

*The whole house is ill*. *We just keep telling our daughter "No*!*"*, *"You can’t*!*" And to my husband*: *"You didn’t lock the fridge*!*"*, *"Who didn’t lock the fridge*?!*"*, *"Give me the keys*!*"*, *"Where are the keys*?! *"*. *(*…*)* (here the name of the child is mentioned—note by researcher K.K.) *took the keys*! *My daughter walks around at night all the time*, *looking for the keys*, *where we have them*, *who has them*. *It happens all the time*, *there’s no peace*, *no such letting up*. *For example*, *today I got up at 4 am for work and she was in my room 4 times during the night*! *I am going to sleep so deeply that she can get the keys out*. *She’s done it so many times*. *She can stand for so long and gently put her hand under the pillow and take out these keys that it boggles my mind*. *And the worst moment is when I wake up and open my eyes*, *her head above my head because she is reaching for the keys*! *She’s very clever despite her limitations*. *And I’m more and more helpless*.

(Interview 14)

The PWS individual’s compulsive search for food forces not just the parents/caregivers but also all other members of the family to remain constantly vigilant and in absolute control. Hence, the consequences of PWS start to affect all the members of the family and the sense of their intrusiveness is directly proportional to the closeness of the given person’s relationship with the patient. The respondents perceive the situation as “the whole family is ill”. The need to subordinate the entire family lifestyle to the requirements of the disease constitutes a source of serious tensions and conflicts. The aspect of the course of PWS related to the impossibility of satiating the individual’s hunger most strongly interferes with all food-related aspects of family communication. Numerous taboos about food emerge in this context. The communicational restrictions in force in the presence of the ill family member must also be complied with by all healthy persons involved: “we do not talk about food”; “we do not ask for seconds”; “we do not say we are full”; “we do not mention our own wishes related to food”:

*When my son eats*, *sometimes I ask a question that I shouldn’t be asking*: *"Son*, *are you full*?*" Sometimes I blurt out something so stupid and he always tells me no*. *He’s never yet said that he was full*. *And then begins begging for another portion*. *(*…*) We make certain arrangements regarding eating and food shopping in a way that prevents our son from hearing*. *It may seem strange*, *but sometimes we arrange what and how through text messages*. *We simply write to each other with our phones in hand*, *at home*, *standing next to each other*. *We write to each other so as not to talk about it*.

(Interview 1)

*We don’t talk about certain things in front of our daughter*. *We don’t talk about the fact that we feel like having something else because we eat the same as*… (the name of the child is mentioned here—note by researcher K.K.). *We don’t talk about what we don’t eat because of her illness either*. *We don’t talk about the meals we eat either*. *We just eat*, *clear the leftovers from the table and lock the kitchen*.

(Interview 13)

*If any of the siblings has a desire to eat a bar*, *chips*, *cookies or something similar*, *I explain to them not to say it in front of*… (the name of the child is mentioned here—note by researcher K.K.). *We only talk about such things outside the home*. *My children have learned this*, *though they have protested more than once*. *There’s silence in the house about food*.

(Interview 15)

The hunger experienced by an individual with PWS becomes increasingly expressive, which negatively impacts the relationship between parents and their healthy offspring–the siblings of the ill person. Food limitations also affect the healthy children, which is a source of tension and conflict. The healthy children feel as though they are being punished for their brother’s/sister’s illness. They fail to comprehend the requirement to adapt to the needs of their sibling. The parents often ignore or misread hunger signals from their healthy children. At the same time, parents who also have healthy children are painfully aware that the emotional consequences of PWS are actually felt more acutely by their healthy offspring, who experience double rejection in the context of the disease. Firstly, the relationship with their parents is significantly affected due to the latter’s permanent focus on the ill child, which results in neglecting the needs of the healthy siblings, not only in terms of parental care but also education or pursuit of personal passions. The second rejection is experienced by healthy siblings in their relationship with their brother/sister with PWS, who, due to the accompanying neurodevelopmental disorders, have serious difficulties in social relations. Here are statements of the respondents that give credibility to the above analyses:

*The calendar is primarily set regarding*… (the name of the child is mentioned here—note by researcher K.K.), *and the consequence of this is that the siblings don’t have everything that they would normally have*. *For example*, *we had to give up on his brother’s extracurricular activities*, *who is 1*.*5 years younger*. *The siblings are always listed in my calendar second*, *unfortunately*. *First there must be room for rehabilitation*, *then there must be room for healthy children*. *And they feel so rejected*, *they even tell me that sometimes*.

(Interview 5)

*Our older daughter was 13 when our son was born with PWS*. *This triggered a crisis in our relationship*, *she was virtually left to her own devices as we became focused on her brother*. *And the disease affected our relationship with our daughter to such an extent that she left home very early*, *moved to another town*, *she was just fed up with the disease and wanted to break free from it all*. *Nowadays*, *we have almost no contact with each other*.

(Interview 7)

*The siblings have begun to treat the illness of (*…*)* (the name of the child is mentioned here—note by researcher K.K.) *as a punishment for themselves*. *I never wanted that to happen*, *but it is*. *The first is the matter of food*, *which the siblings have never had as much of as they want because everyone at the table got the same portions*. *Sometimes they got up from the table hungry*, *had to eat in secret*. *They just wanted to eat normally*, *like their friends do*. *But they didn’t reject him*! *It is the other way around*. *And here the second thing is that PWS is also a spectrum of autistic behavior*. *Our ill son often rejects*, *pushes away people who care for him*, *care about him*, *want to be friends with him*. *And he does the same with his siblings*. *It is a very difficult relationship*.

(Interview 2)

The constant control over the PWS individual’s obsessive search for food can sometimes become an obsession for the caregivers themselves. As the scope and restrictiveness thereof continue to grow, so do the emotional costs. This has a particularly strong effect on the mothers who usually bear most of the responsibility of managing the consequences of the illness. They experience exhaustion and helplessness in the face of constantly growing unpredictability, but also the ruthlessness of their children’s efforts to acquire food. The mothers give up their careers and take on the role of the child’s “satiety centre”, a task in which they are usually forced to cope with alone. Faced with a sense of isolation in their care for the ill child/adult, with the constantly mounting responsibilities and clearly formulated expectations voiced by the father, the mothers confessed to experiencing depression and anxiety, often strong enough to require psychiatric and/or psychological intervention:

*There was a time when I simply could not handle it anymore*. *I felt dejected*, *didn’t even feel like making dinner*. *I let myself go*, *didn’t wash my hair*. *I cried a lot*, *eventually I had to see a psychiatrist who diagnosed me with depression*.

(Interview 8)

*I constantly have to struggle to maintain a sense of normality*. *My husband’s expectations are very high*, *he would like things to be normal*, *he expects too much of me and our son*. *And it’s simply impossible*. *I can’t do it*. *We never talk about the disease*. *I read a lot about it but when I find something interesting and want to share it with my husband*, *I have to force him to listen so he is aware that such things can happen in this illness*. *I went through a crisis when I had no more strength to control this hunger*, *I couldn’t handle the organization*, *I had no patience*. *Because there can be no normal life with this illness*. *We get nothing from this disease*, *and it drains everything from us*.

(Interview 10)

*This illness of my daughter has made me so exhausted that I myself have problems with being overweight*, *I have problems with everything*, *with my husband*, *with my daughter*. *Suicidal thoughts appeared*. *I was thinking of killing my daughter first*, *then myself*. *(…) I fell into quite a deep depression*, *I gave up*. *I don’t have the strength to believe that things can be better*. *Some old fears returned*. *I think my husband has never come terms with the fact that he’s an ill child*. *And this is probably the most difficult for me*. *For about 8 years I have started noticing that I feel bad about it*. *My husband isn’t up to looking after his ill daughter*. *I am always alone*, *alone*, *alone*. *I have to look for solutions*, *I have never had any support from him*, *so that we could do something together for our daughter*. *I think I made a mistake that I became so involved in taking care of our daughter from the very beginning*. *There’s no support in my family*, *there’s denial of the problem*. *So*, *as soon as I deal with my depression with medication*, *I’m going to get a divorce*. *I’m going to go back to work*. *My husband doesn’t help me in anything*, *I don’t have the support I need*, *that a woman needs from a man*. *I keep hearing*: *"You’re strong*, *You’ll be fine*.*" I’m not strong*, *I’m not strong anymore because I’m depressed*.

(Interview 14)

The severity of the described crisis experience is reflected in the resignation expressed by some of the caregivers, their (ultimately not realized) intention to simply allow the patient to eat without any limitations.

### III. Crisis in response to the social milieu’s failure to understand the nature of the illness

The next stage in the caregivers’ PWS illness experience emerges unexpectedly, once the parents have finally managed to accept the lifestyle changes imposed by the illness and gain some measure of control over its consequences, as well as begin to see tangible results of the integrated child therapy. The crisis stems from the relationships with the family’s social milieu, whose members fail to understand the nature of the disease or the patient’s needs that result from the same, and therefore are unwilling to provide the family with the support it requires.

The experience of this stage of crisis is primarily marked by the caregivers’ feelings of overwhelming loneliness and helplessness, which force the family into self-imposed isolation. A significant part of the problems emerging in the family’s social relationships is closely related to the behaviour of members of extended family circles, primarily the grandparents. A sentiment shared by many parents of PWS children/adults is that “the greatest enemy of a person with Prader-Willi syndrome is the grandmother”. This was confirmed by the respondents’ accounts, which identify the grandparents as the persons most severely interfering with the patients’ dietary regimes and therefore the efforts made by the caregivers. The grandparents tend to act based on their lay, non-medical understanding and interpretation of PWS as a condition of constant hunger, which compels them to “cater” to the patient irrespective of the established feeding regimen because they feel hungry. When left in the care of grandparents, the patients often find themselves in places with open access to food, are offered sweets, are as well as sometimes encouraged to question the rules imposed on them by their parents:

*As a parent*, *during all social events I am forced to not only control my child but also all other members of the family*, *everyone sitting at the table*, *especially the grandparents*. *For this reason*, *we are perceived as overly strict in our methods*, *the grandparents keep telling us that we are terrorizing our own child*.

(Interview 2)

*It’s a constant problem—grandparents*, *who here and there smuggle sweets*, *feel sorry for the child and disrupt the entire rhythm of nutrition*. *As for our grandparents*, *they destroy all our upbringing*, *our principles that we have introduced when it comes to nutrition*. *We have such rules that our daughter can’t drink sweet beverages*, *for example*, *and her grandmother gives her them all the time and says*: *"Give her a break*, *give her a break*.*"*

(Interview 13)

*Grandma only recently understood the true nature of the illness*. *I had to struggle with her for years to stop her from giving my son food*, *sweets*, *money*. *My son is now 18 and my mom did not understand the disease until he was 17*!

(Interview 10)

The beliefs of lay people in the extended social milieu of the family stand in direct conflict with the professional definitions of PWS, to which caregivers of PWS individual’s subordinate their lives. Thus we are dealing with a clash of two distinct interpretative perspectives: the lay and the professional. However, the uniqueness of the situation lies in the fact that both of them function within the lay referral system of individuals with PWS. Common-sense interpretations of the illness, emphasized overbearingly and dangerously to the sick child by his/her grandparents, interfere with the parents’ ‘illness work’ and may lead to harmful separation of the family from their social environment and are a source of conflicts. In effect, the families experience social isolation. It can be reasonably concluded that the same stems from the caregivers’ inability to effectively educate friends and family members on the behavioural specificity of the condition. Hence, the effort invested in creating the family’s new social identity adapted to the requirements of the illness may be perceived as futile:

*People just don’t understand how a child can be constantly hungry*. *Nobody believes me when I tell them my child can eat food from the garbage*, *they think we*, *the parents*, *are exaggerating or downright lying*. *As the family of a child suffering from this syndrome*, *we feel socially isolated and lonely*, *in fact we are left to our own devices and must cope with all of it alone*.

(Interview 5)

*We have become recluses because others*, *even close family members*, *cannot understand the condition*. *They listen to us talking about our eating rules and then laugh*. *We have isolated ourselves because of it and no longer maintain contact with our families*.

(Interview 9)

*The worst thing about this illness is the lack of understanding from others*, *teachers*, *therapists*, *pretty much anyone other than us*, *the parents*. *I would expect at least the people working with my son to grasp the nature of the condition and I cannot understand why they don’t*.

(Interview 10).

The interpretations of the family’s situation formulated by its social milieu tend to question the validity of the medical diagnosis of PWS. Some members of the extended family find it difficult to accept the description of the illness provided by the caregivers or openly express doubts about the diagnosis. This is due to the fact that PWS individuals often do not show the stigmatizing bodily deformities that would be immediately apparent to a lay person (of the 13 PWS individuals under the age of 18 and living under the care of the respondents, only one was found to be slightly overweight), while at the same time the PWS individuals’ social milieu constantly observes the strict “no free access to food” policies imposed by the caregivers. In the eyes of a lay person, a slim child hardly fits the clinical description of PWS. They fail to understand that the child’s correct body mass is in fact evidence of the effectiveness of the caregivers’ extensive work. In this context, caregivers encounter difficulties in obtaining social legitimization of their efforts aimed at controlling their child’s food intake that will have to be continued for the rest of the PWS individual’s life. This also leads to difficulties in obtaining financial support from dedicated institutions:

*My child is slim*, *in fact he looks better than many children in the healthy population*, *with so many kids now who are overweight or obese*. *But it is all thanks to our hard work*, *we give him growth hormones*, *maintain dietary regimes and that is why our child is slim*.

(Interview 1)

*Nobody sees my child as an ill child*, *because my child is slim*, *well-behaved and at first glance you can’t see that he is ill*. *My child doesn’t require 24/7 care either*, *I don’t have to change my son’s clothes*, *wash or change his diapers*, *just like parents of children with cerebral palsy*. *But I have to have 24/7 control over him*, *24 hours a day I have a head full of whether my son will eat something or something has been left out*. *I keep thinking how many calories he can eat*, *or how many he’s already eaten and what he can possibly eat*. *It is a continuous*, *never-ending story that goes on all the time*, *whether it be day or night*, *morning or evening*, *or right after or before meals… It’s the same thing in your head all the time*. *He’s hungry all the time and looks for that food all the time*. *(…) We’re perceived as parents who are simply cruel*. *I am perceived as a crazy mother*, *our child looks good*, *so I often hear*: *"If you allowed him more freedom once*, *nothing would happen*.*" Not only do I have to deal with the fact that my child hates me for how we function because my son told me that*, *but I also have to face an environment that completely doesn’t understand the child’s illness and doesn’t understand me in all of this*.

(Interview 2)

*My son is in excellent shape*, *he goes to high school*, *to an integrated class*. *He swims*, *cycles*, *reads*, *has passions*, *keeps slim*. *I cannot even try to apply for some aid from a foundation because they want you to post a photograph of your child and ask for money*. *We could really use it for the rehabilitation*. *But who will believe my son has Prader-Willi after looking at his photo*? *They’ll just call me a fraud and I’ll be inundated with internet hate*.

(Interview 10)

Some bizarre situations take place between the parents and paediatricians examining their children. It happens that, given the absence of the morphological symptoms of the disease, physicians actually question the validity of the PWS diagnosis. From a sociological perspective, we have an extremely interesting situation in which the medical interpretative perspective is employed by lay people who consequently find themselves in conflict with medical professionals employing, paradoxically, commonsensical interpretations. Thus, Freidson’s clash of interpretative perspectives, lay and professional, is reversed:

*I believed the diagnosis*, *but when our son was hospitalized in the regional hospital*, *the paediatrician insisted our son did not fit the illness description at all because he was slim*. *He tried to convince us that it must have been a misdiagnosis*, *that our son did not have Prader-Willi*. *My husband clung to that false hope*. *But I didn’t because we had already done the genetic tests in Wrocław and Warsaw*.

(Interview 9)

*My (*…*)* (the child’s name is mentioned here—note by researcher K.K.) *is 8 years old*, *a very slim boy*. *When I go to the pediatrician and say that my son has PWS*, *the doctor says*: *"Impossible*, *he is slim"*. *I then answer*: *"Yes*, *he is slim*, *but he’s got PWS"*. *I have heard this many times already from pediatricians with whom we need to consult*, *for example*, *on vacation*.

(Interview 5)

*When we go to the first better pediatrician*, *it has happened more than once*, *that he looked at me like a crazy mother*. *And I have to explain this illness to him because doctors say that if my son is slim*, *even thin*, *he can’t have PWS*. *The pediatrician doesn’t have the slightest idea about this*.

(Interview 1)

The families’ self-imposed isolation also stems from stigmatization, perceived by the caregivers as unfair and harmful, triggered by the social milieu in response to the behavioural disorders of the child, among others, fits of anger and rage, verbal and physical aggression, compulsive behaviour (including hoarding and compulsive sexual behaviour). All these behavioural problems are indicated by the respondents (particularly in cases of children diagnosed with type II PWS, i.e. disomy) as the most burdensome symptoms of the illness, far worse in fact than the insatiable hunger or obsessive search for food. Stigmatizing judgements formulated by others, alluding to “an ill-mannered child” or “inept parenting” further deepen the family’s isolation. Tantrums experienced by the children in public, which are beyond the control of the patients themselves as well as their caregivers, frighten people. The tendency to use vulgar language, to insult those trying to offer help, effectively discourages people from the social milieu to maintain any contacts with the family. It is observed that it is primarily due to the children’s behavioural problems that the caregivers continue to make efforts in their social environment with the hope of replacing the lay understanding of the child’s problems with a proper, medical definition:

*I have already given up trying to explain our eating principles*, *but when I saw how people react to my son’s rages*, *that people become scared of us*, *I started to explain*, *tried to make them understand*. *But the people around us do not want to talk about this illness*, *they run away*, *break off contact*. *When we talked about the doctors*, *visits*, *procedures*, *our failures*, *people would downright say*: *“that’s the last thing I want to hear about*!*” So*, *we have limited our contacts with others*, *even with family*.

(Interview 5)

*I can’t control my daughter’s aggression*, *we haven’t been able to deal with it since the first attack and we still don’t know how to deal with it*. *We moved out to the countryside so she can yell here*, *sometimes she goes outside and yells for 40 minutes*. *When he gets into such a rage*, *she marches upstairs*, *slams the door*, *smashes anything possible*. *There’s nothing left in her room*, *not even a bed*, *only a thick mattress*, *a TV she never smashed*, *and a wardrobe*. *I bought a two-door one from Ikea for 200 zlotys because she smashed all the previous wardrobes*. *So I buy one for PLN 200*, *so I don’t regret it*. *And that’s all she’s got in the room*. *There’s nothing to smash there*, *so she goes there and beats what she’s got*, *lies on the bed and cries*. *(*…*) When my daughter has such attacks*, *I just sleep for 4 hours after her attack*, *it exhausts me mentally*, *I don’t have the strength to stand on my feet*, *I have to lie down and sleep with her*. *But I repeat to her*, *I tell her*: *"If you hit me*, *I’ll hit you too*.*" I also try to cope with that*. *(…) I have sisters*, *I have siblings*, *but they all live their own lives*. *Nobody thinks I may need some support*.

(Interview 14)

*The behavior related to sexuality in this illness is shocking*. *It’s strange to me*. *At one point*, *I noticed that my son began to masturbate*. *Only he started doing it in front of our eyes*. *When he got upset*, *he would lie down on the carpet*, *start crying and masturbating*. *Also in public a few times*. *He takes a hairy sweater from the wardrobe and pushes it into his crotch*, *and I think it gives him pleasure*. *It was so frequent and burdensome for us that we made a separate room for our son at home*, *to which he went for this purpose if he needed to*.

(Interview 15)

*The attacks are becoming more frequent and severe*. *At the end of the school year*, *our son fell into such a frenzy at school that he yanked the hair of the support teacher he’s got there*, *called her the most profane names*, *he’s got a high level of aggression*. *The same happened to me lately*… *when he fell into this frenzy*, *I didn’t even know when he grabbed my hair*, *started to pull me*… *and he is only 9 years old*. *What I fear most is what will happen when he is 15 or 18 years old*, *what this attack of madness will look like*. *This frenzy is getting bigger and bigger*. *It lasts up to 45 minutes*, *there’s also drooling and slamming*. *Once*, *he was even able to grab my husband by the shirt*. *(*…*) My mother*, *brothers and sisters-in-law don’t understand that (*…*)* (the name of the child is mentioned here—note by researcher K.K.) *is ill*, *they don’t think about it at all*. *I explain to them*, *I expound this illness*, *but they don’t understand it*. *They don’t understand and are afraid of this illness and us*. *I no longer have the strength to explain these attacks of madness all the time*. *Neither our closest family*, *nor neighbors*, *nor friends understand it*. *Therefore*, *our contacts with them are limited*. *We live our own lives*.

(Interview 9)

The unpredictability of the occurrence of explosive disorders, the variable duration and intensity of such fits mean that parents find themselves unable to temporarily entrust the care of their ill child to anyone else. Consequently, they experience a permanent lack of spare time, which is another significant burden for the caregivers of PWS persons. It has a negative impact on their mental health and they also have problems in the spheres of professional, marital and sexual life, oftentimes forcing the parents to give up on social life or any form of entertainment.

Analysis of the empirical data indicates that the PWS experience is an interactive phenomenon affecting the social relationships of the patients and their caregivers not only in the context of their families, but also any other person the patients come in contact with in their everyday lives. The caregivers’ accounts repeatedly indicate that the sphere of social interactions is the aspect of everyday life that suffers the most as a consequence of the illness. Contacts with family members and friends are devoid of spontaneity and cannot be maintained in traditionally accepted ways, particularly in terms of access to food. The related restrictions, stemming from the need to continuously work on the effects of PWS, and the treatment of any communicational messages related to food as taboo, mean that the group of people maintaining social contact with the family of a person with PWS gradually narrows. The intrusive eating restrictions, socially awkward tantrums, publicly displayed compulsive sexual behaviour (e.g. uncontrollable masturbation), and finally incidents involving the theft of money or valuables, become the reasons for the conscious decision about (self) isolation of the family of a PWS person.

### IV. Crisis in response to attempts to plan the future of PWS individuals

In our research, we were able to demonstrate that while the caregivers of younger children with PWS perceive time in strictly crisis-oriented terms, living their lives “here and now”, as the child grows, the uncertainty about what the child’s life will look like in the future and who will provide them with the needed care after the parents’ death gains dominance. The caregivers are aware that the PWS individual will never be able to lead an independent life, hence steps are taken towards their legal incapacitation. This issue becomes the central theme of the next stage of crisis experienced by caregivers of people with PWS. Fears and anxieties arise when attempting to plan the future of a PWS individual (who is no longer a child). Most caregivers form distinctly pessimistic visions of the PWS individual’s future, rejecting optimistic scenarios as unrealistic. As they are unable to imagine their child living an independent life in the future, even before the individual reaches adulthood, they take the drastic, in their own assessment, step to declare their son or daughter legally incapacitated. In the context of that decision and the actions involved, they are forced to proceed intuitively and without any outside support, based solely on their own judgement of the situation. It should be noted at this point that while in all other aspects of looking after the PWS person the caregivers may count on some measure of support from various self-help groups, when it comes to legal incapacitation no such aid is provided as even in caregiver circles the issue is highly controversial, evokes extreme emotions and leaves these circles divided:

*I have to deal with this issue intuitively*. *Trying to imagine my son’s future*. *No one will tell me what I should do as a parent of a PWS child*. *Even other parents who have the same problem*. *Legal incapacitation is another stage of the illness and even though I’d learnt a lot about it*, *I still don’t think I was prepared for it*. *It was a drastic step*, *but we went through with the process of declaring our son incapacitated*. *It was the worst stage of the disease; I’ve been ill ever since because of it*. *I somehow managed to get through the official incapacitation process*. *But when the court announced its decision*, *it was terrible*, *it was so final… That was such a horrid experience that I haven’t been able to pull myself together for weeks now*, *even though it was done with full awareness and full responsibility*. *I knew it had to be done but that didn’t make it any easier*. *It was really difficult*, *the final act of it all*.

(Interview 10)

*I think about this incapacitation all the time*, *but I know I have to do it*, *although for now I’m strongly putting off*. *Because if we don’t*, *I know someone might take advantage of it*. *My son will sign everything*. *If someone told him*: *"Sign me here*, *you’re a nice boy*, *I’ll buy you a good dinner and I’ll buy you something sweet"*, *I think that he will sign everything that someone suggests to him*. *I have to incapacitate him because I won’t find peace*.

(Interview 15)

*The situation in which an adult "prader" isn’t incapacitated is a curse for himself*. *I will do it in a moment because we already have an application*. *Our son has recently turned 18*. *As an association helping people with PWS*, *we’re now intervening and helping in a situation where the parents haven’t incapacitated their son*, *who turned 18*, *and he’s been hired by McDonald’s as a cleaner*. *He got his first paycheck*, *all of which he’d eaten*. *He’s got access to his parents’ account*, *ran away from home and said he didn’t want any contact with his family*, *and that he didn’t wish anyone to inform the family where he was*. *He said he was not coming home because he was free at last*. *Social assistance put him in a night shelter*. *In the Prader-Willi syndrome*, *a person can’t function independently*, *even if he’s in the intellectual norm*. *Sooner or later it will end up with imprisonment or premature death*. *In our association*, *there are also divided voices on the issue of incapacitation*. *I know that if my son isn’t incapacitated*, *he will also turn away from us*.

(Interview 1)

Legal incapacitation allows the caregivers to prevent the PWS individual from proceeding with the plans and threats articulated over the years of struggling against the imposed dietary and behavioural restrictions. The biographic project of a PWS individual tends to be heavily marked by plans to take up some form of employment and thus realize the lifelong dream of indulging in an uncontrolled diet. Because the parents are perceived as a primary obstacle to realizing such aspirations, persons with PWS plan to move out of their family home and sever contacts with their caregivers as soon as they come of age.

The caregivers’ experience remains, nonetheless, marked by a strong fear concerning the PWS individual’s life after the death of the parents; that they will be left to their own devices, deprived of the support of people capable of providing them with professional care. Caregivers do not count on institutional aid in this respect as the specificity of PWS prevents committing the individual to standard care facilities due to the open access to food products. Another factor escalating the caregivers’ anxiety is the lack of institutional facilities tailored to the specific needs of individuals with PWS, offering comprehensive and integrated care. This leads to overwhelming feelings of defeatism and fatalism:

*We talked with my husband*, *what will happen later*, *in the future because we are afraid what will happen to our son*, *what will happen next*??? *Once we’re gone*, *he will be all alone*. *He will be alone*, *so he will steal*, *eat from the garbage*.

(Interview 8)

*There is no alternative for my daughter’s future*. *Which is why I would prefer to bury my child*, *I’d rather she died before me*.

(Interview 6)

*I don’t know where I can put my son to keep him safe*. *I don’t know who could take care of him later in life*, *when I’m old and I won’t be able to cope on my own*. *I am afraid that some illness will come and I won’t be able to look after him anymore*. *Each of the siblings will start their own family*, *they will have their own children*, *so they won’t be able to devote themselves to him*. *Besides*, *I can’t stick them with it*. *They love him very much*, *I can’t say they don’t*, *but I can’t blame the kids if they can’t help us*. *Although I know it would be best for my son to stay in the family*. *It should be like that*, *but I have no conscience to ask for it*. *Looking realistically*, *nor is there any place or facility anywhere where he could be placed*. *There isn’t*. *There’s no place on earth for my (*…*)* (the name of the child is mentioned here—note by researcher K.K.).

(Interview 15)

*I don’t see any future for my daughter but by my side*. *I don’t know what would happen if I were to die earlier*. *I don’t see our future at all*. *I will get older with my daughter*, *but I don’t know what it will be like*. *And God forbid when I’m gone*. *I don’t expect that my healthy daughter will take care of (*…*)* (the name of the child is mentioned here–note by researcher K.K.), *that she will take care of her as a sister*, *that she will take her in because I know that it is a difficult illness*. *I know that it would destroy the life of this healthy one*. *But I also made it clear to my healthy daughter that if anything happened to me*, *no one had the right to send her* (the daughter with PWS–note by researcher K.K.) *to any center*.

(Interview 14)

When pondering the organization of care for the PWS individual after one’s death, caregivers commonly experience a moral dilemma regarding placing that burden on the healthy siblings. They tend to ask themselves: “Do I have the moral right to expect that of my healthy child?”. In the context of the anxiety related to the fate of the ill child/adult an internal conflict unfolds. The parents feel torn between the conflicting interests of their healthy and ill offspring; they try to justify their efforts to assign the duty of taking care of the ill individual to a healthy sibling for the good of the ill child. As follows from the conducted interviews with the surveyed caregivers, that conflict seemed to be not fully resolved in the time period under scrutiny.

The experience of this crisis stage also entails the usually unsuccessful interpretative work aimed at finding some meaning and value in the illness. None of the caregiver accounts indicate any noteworthy benefits that might stem from the illness and its course. None of the respondents see their PWS experience as having any existential value, somehow stimulating personal growth, yielding any benefits whatsoever for the people involved:

*I cannot say that the illness has brought anything positive into my life*. *It does not bring together*, *it divides*. *I cannot be thankful for any of my son’s achievements in this illness*, *for any progress made because it remains the progress of an ill rather than a healthy child*. *And I wish he was healthy*. *This illness brings about such difficult situations*, *forces me to make some choices and decisions—I had to quit my job*. *I always look*, *I am a very positive person and I always look for the positive side of an event*, *I always see its purpose*. *I think nothing happens in vain*, *but not illness*! *I do not think that illness leads to anything good*, *an illness will always be an illness*. *There’s no point in making it into something it isn’t*.

(Interview 10)

*I don’t see any benefit from this illness*, *I think that if someone could change it*, *I wish it would disappear from our lives*. *This illness is difficult for the ill person and for the whole surroundings*. *We do everything to make our son live as long as possible*. *But in our association*, *five or six children died of obesity and obesity complications*. *So everything we do*, *we do so that he lives as long as possible*, *although the doctors told us that we will bury our child and I live with such a thought that I will bury him*. *I don’t see the point in it*.

(Interview 1)

*This illness is very burdensome*. *We have been with it for 30 years*. *And although we’ve learned to live with it*, *I don’t see any of its positives*. *There are no positives here*! *This is constant suffering*! *We’re an abnormally functioning family*. *No one will understand it unless he’s experienced it himself*.

(Interview 19)

As perceived by the caregivers, PWS is unequivocally a deviation as it radically upsets both the patients’ and their families’ capacity to function normally. At no point in the course of the disease, despite temporary periods of regaining a measure of relative stability having coped with the experiences of subsequent crisis stages, are the caregivers able to return to normal activity patterns of people belonging to the world of the healthy. PWS becomes an integral element of the caregivers’ everyday lives which tend to be dominated by the illness and never lose their deviational character. The essence of the PWS experience seems to entail a complete change of one’s entire way of life to an extent that removes it far from the experience of parents with healthy children. The constant presence of the illness radically disrupts the functioning of the whole family, prevents any semblance of normality, in the absence of any subjective efforts aimed at adapting to the condition on the part of the patients themselves. At this point, however, we wish to make it clear that the foregoing observations apply only to the period of experiencing PWS that was the object of study. There is no reason to exclude the possibility of occurrence of adaptive changes in the further course of the process of experiencing PWS by caregivers of persons with this condition.

## Discussion

The primary aim of the study was to discover and describe the specifics of the illness experience of the caregivers of PWS children/adults and identify its key elements. The description of the specificity of the process of PWS experience presented in this publication facilitates a better understanding of the nature of the illness experience from the perspective of caregivers of individuals with PWS, which may prove valuable in developing means of systemic support for such families that would take into account the specificity of the phases in the course of PWS.

The primary findings of the presented research pertain to the processual character of the experience of caregivers looking after PWS individuals. The leading interpretative category emerging from the interviews is one of crisis: the surveyed familial caregivers interpreted their own experience related to their child’s illness primarily in terms of a crisis unfolding against the background of “the illness work”, which forced them to reconstruct the family’s entire way of life. A classic, pioneering analysis with a parallel profile was conducted in medical sociology by Davis who, in the 1950s, based on a series of interviews conducted with parents of children with spinal paralytic poliomyelitis, developed a model of the crisis experienced by such families. Subsequent crisis stages were distinguished in the model. [30: p. 20–44]. In Davis’s research the emergence of the respective stages in the course of the illness was triggered by the occurrence of specific events (*clues*). Strauss et al. [35] also observed that specific phases can be identified in the psychosocial trajectory of a chronic disease.

In the authors’ reported own research, we also tend to conceptualize the obtained research results in terms of phases of the illness experience process, but we do not suggest that linear movement of individuals through indicated phases is a universal pattern of experiencing PWS. The logic of presenting the results of our research does not have to fully coincide with the logic of the experience of a crisis in all the caregivers of individuals with PWS. In using linear logic, we wanted the experiences of the respondents to be more understandable to the recipients of this content. “However, experience–as K. Charmaz writes–does not have to have a linear structure, and its boundaries are not always clearly marked. For example, the experiences of an illness, and even less its consequences, are not always in line with the linearly progressive process created by the author.” [31: p. 173] In this context, the cited author refers to the publications of Anselm Strauss and Barney G. Glaser [33, 36] who, in their classic works on grounded theory, insist on “discovering and analyzing a basic process, which may not work for you” [31: p. 173]. We are also inclined to say after K. J. Doka, that “*the use of [the term] phases emphasizes that individuals have to cope with distinct challenges and issues at different points in [*…*] illness*.” We also assume that “*the phases simply represent different points in the ways in which many individuals experience illness*” [37: p. 87].

The analysis of our own empirical material yielded a four-phase process of the illness experience where the respective phases of the crisis manifesting in the lives of the surveyed families varied in terms of the duration and intensity. Strauss suggested that the specifics of the particular phases of the psychosocial trajectory observed in the course of an illness are determined by the biomedical characteristics of the respective stages of the disease as such, which define the closely correlated actions undertaken by the patient and people in their social milieu. Similarly, in our own study the biomedical trajectory of PWS determined the specificity of the illness experience in the families of PWS children. Analogous findings were made in a study by Chaij et al. [38], where it was demonstrated that both the family dynamics and the specificity of the challenges faced by the family depended on the child’s age and progression of the illness, while any ongoing stressors in this regard tend to require new adaptive efforts.

The available empirical data documenting reactions to various medical diagnoses indicate, with due consideration for the clinical character of the given problem, a wide spectrum of reactions, from shock, through disbelief, to a sense of relief stemming from the eventual diagnostic classification of the experienced health problem [8, 39, 40]. Corbin and Strauss [8] use the term “*diagnostic limbo*” to draw attention to the period of being “*suspended in time*” while awaiting information to verify one’s expectations or fears regarding the ill person’s condition. At the same time, they emphasize that the full reception of the diagnosis consequences may be delayed in time. In Davis’s study, a characteristic element of this stage in the course of the illness was the feeling of helplessness and personal loss. The parents of the ill children “broke down” upon receiving the diagnosis, while their imaginations started to evoke the likely scenarios of the disease’s progression (crippling, iron lungs, etc.) [30: p. 32]. An interesting aspect of Davis’s findings concerned the clash of lay and medical interpretative perspectives in the process of reaching the medical diagnosis. While in the pre-diagnostic stage the child’s health problems were interpreted in the commonsensical perspective, the diagnosis marked a point from which the interpretative perspective usually shifted, and the medical approach became dominant. Davis states that, in general, the interpretational activity of the parents had usually not completely extinguished, as the caregivers continued to oscillate between the medical diagnosis of *polio* (i.e. the medical frame of reference) and various commonsensical interpretations [30: p. 23–29]. In this context, Davis used the expression “<*shopping around> for information*”, underlining that the process occurs both within and outside the hospital environment [30: p. 59, 61].

In our own research, interesting phenomena were observed, which consisted in the fact that the parents operated steadfastly within the medical interpretative framework and even acted as its advocates, while the family’s broader social milieu, and even medical professionals not familiar with the PWS problems often relied on commonsensical, non-medical interpretations in their assessment of the PWS individuals’ health situation. In this context, should we recall Elliot Freidson’s concept of the clash between lay and professional interpretative perspectives with regard to medical treatment [41], we would discover that in the context of the PWS experience, the roles of the actors partaking in the conflict were reversed, while “clash of perspectives” itself took place entirely within the “lay referral system” [41].

The available literature on the subject describes the leading behavioural characteristics of PWS, which to the greatest extent generate the emotional burden of caregivers. Mackay et al. [2] mention “*food-seeking activities*, *restrictive or repetitive behaviours and difficulties with social communication and reciprocity*”, considering them as the main elements of the PWS behavioural phenotype that generate significant consequences for the maternal and family well-being. Sarimski [3] indicates the insatiable appetite as the primary phenotypic feature of PWS children. In the study by Mazaheri et al. [25], it was noted that the most significant source of burden affecting the caregivers as well as the patients’ healthy siblings is the multidimensional character of the behavioural manifestations of PWS that “surround” the primary problem of “diet management” and significantly hinder control over it [42]. Griggs et al. observe that the hyperphagic phenotype characteristic of PWS is amplified by intellectual disability, obsessive-compulsive disorder and/or autistic behaviour [14]. The level of caregiver stress is further increased by the fact that the simple, everyday activity of eating constitutes a life-threatening risk for PWS individuals [25]. A additional burden for caregivers is the acute awareness that the level of dietary control is a determinant of the PWS individual’s health and life expectancy [38]. In the group analysed in the present study, the vast majority of patients genetically diagnosed with PWS were neither obese nor overweight, i.e. they lacked the primary phenotypical symptom of PWS (80% of the patients; in the group of 13 patients under the age of 18, only one was slightly overweight), which indicated that in most of the cases, the caregivers were able to implement effective dietary control strategies to protect the children from complications common for the illness. We are therefore dealing with a “success in the illness” in the biomedical perspective, which, however, does not mean that the disease was perceived as an experience in any way existentially meaningful or significant. In the eyes of the parents, the condition does not cease to be a burdensome deviation that deeply distorts the functioning of the family. Any efforts made with the aim of normalizing the illness remain futile in the face of its destructive impact on the social interactions and everyday lives of the families.

The unique character of this study stems from the fact that it emphasises the “tangible” effects of the caregivers’ efforts (80% of the PWS individuals maintained normal body weight), which may be treated as a marker of successful management of the illness by the people looking after the PWS individuals. It also documents the long-term sense of crisis and the deviational nature of the psychosocial consequences of PWS that are not subject to normalization, nor can be attributed any existential meaning (in a time period under scrutiny). For instance, in the study by Allen [42], the researcher identified the primary practices of “body management” employed by families providing care to PWS individuals (“restricting access to food”, “keeping occupied”, and “use of routine”), but did not specify whether the same were applied by effective or ineffective caregivers, or whether they succeeded in protecting the health of the PWS individuals.

The present study identified the primary source of caregiver emotional burden as the necessity to constantly control the eating habits of the PWS individuals, which proves problematic once the child gains locomotive independence and becomes able to eat and acquire food without help.

It is noteworthy that our study, based on interviews conducted with the caregivers of twenty individuals with PWS up to 23 years since the original diagnosis, did not confirm the occurrence of the 4^th^ stage in the course of PWS as described by Miller et al. [11], specifically the one in which the PWS person regains the ability to feel satiated. In a study by Miller et al. conducted in a group of 82 people genetically diagnosed with PWS, this phase was identified only in two adult patients [11]. Griggs et al. [14] reported the existence in the natural course of PWS of the 4^th^ stage characterised by a decrease in hyperphagia occurring after the age of 30, but were unable to identify the causes of the said change [14]. They suggest that the identification of such individuals and their inclusion in research may set the direction of future research on PWS [14]. Assuming that in the case of all the individuals included in the study by Miller et al. the genetic diagnosis of PWS was sound, the low incidence of this variant in the natural course of PWS may be the reason why no such case was encountered in our own research, hence preventing us from documenting the change in the caregivers’ experience resulting from the radical shift of the illness’s biomedical trajectory. Whittington et al. [43] expressed reservations as to whether the weakened manifestation of the behavioural phenotype upon reaching maturity, after its intensification in the periods of adolescence and early adulthood, constitutes a sufficient basis for its classification as a separate phase of PWS. When taking into account the entire process of the caregivers’ PWS experience, we are most certainly faced with a situation in which the “*physiological unfolding of an ill person’s disease*” [44: p. 64] is effectively modified as a result of the caregivers’ active efforts to that end, in a way that in most cases prevents obesity in PWS individual and its related complications. From the sociomedical perspective, it should be pointed out that the description of the “*total organization of work*” inspired by the illness, with particular consideration for “*the impact on those involved in that work and its organization*” [44: p. 64] developed in the course of this study, serves the roles attributed to research on illness experience within the interpretative interactionist paradigm.

The review of literature pertaining to this subject matter revealed that in order to minimise the PWS individuals’ exposure to food, caregivers may limit their social interactions and those of their entire families [2]. In our own research we established that an important factor leading to the limitation of social interactions is the lack of understanding of the challenges entailed in looking after a PWS individual by the surveyed family’s social milieu. In particular, other people tend to perceive the eating restrictions imposed on persons with PWS as excessive. This is particularly true (as follows from our findings) if the PWS individual maintains correct body mass, which seems to justify, in the eyes of people from outside the family, lifting the dietary restrictions. This issue was also discussed in a study by Chaij [38], in which some of the accounts of mothers mentioned a lack of understanding and support, or the formulation of hurtful comments by people in their social milieu who questioned the need to control the child’s dietary habits. It seems that such attitudes are strengthened by the culturally ingrained tendency to treat food, on the one hand, as a type of reward, and on the other, as a form of vicarious, at times compensative care for a child that ought to “look well”. Dietary restrictions consistently upheld also outside the family home are perceived in this context as an overly severe and unjustified form of harassment, particularly if the child is not obese. The caregivers of PWS children repeatedly described situations where people from their social milieu are able to directly infringe on the established rules by feeding the PWS children, thus destroying the caregivers’ painstaking efforts [45]. The problem was also mentioned in our own study where the parents commented on the behaviour of the children’s grandparents.

Planning the future by the caregivers looking after PWS individuals is focused on maintaining the lifelong care policy of “no free access” to food as the individuals remain permanently unable to make independent food-related decisions [14]. This necessitates planning the future of a PWS individual in such a way so as to ensure routine and tight control over their eating habits are maintained. In our research, such issues, leading to serious ethical dilemmas, proved to be the determinants of a separate, specific crisis stage in the course of the caregivers’ PWS experience. They seem to constitute the most difficult problem to solve in the context of looking after a PWS individual, given the absence of institutional solutions dedicated to these persons.

In the final phase of the discussion, we would like to refer to the unequivocally negativistic colouring of the recounts of the caregivers of individuals with PWS found in our research, located on the opposite extreme of the vision of "being successfully ill" reported in the literature on the subject, describing living an active and meaningful life in the context of chronic illness [46]. The results obtained by us do not seem to be a function of the caregivers’ depression, as evidenced by the presence of issues of psychological support or treatment of depression in only two interviews, as well as the high effectiveness of the caregivers in the field of instrumental care for individuals with PWS, expressed in the vast majority of cases by their normal body weight. We rather interpret them as a function of contextual determinants, which, as we presume, may be related, on the one hand, to the cultural strategy of the perception of illness and being ill as a life tragedy, and, on the other hand, may be a function of the institutional and functional failure of the medical system in Poland, within which there is to date no subsystem dedicated to the treatment and support of individuals with PWS. There are no support institutions that would provide these people with professional, round-the-clock care taking into account the specificity of the syndrome, i.e. with no open access to food. Professional activation of this group is impossible because no sheltered employment establishment has been established in Poland to date, in which individuals with PWS would be provided with nutrition control. A very important factor that can explain the physical and mental overload of the caregivers is the lack of a coordinated health care system for individuals with rare diseases in Poland, centers and specialist clinics where patients and their caregivers could obtain comprehensive assistance. The presented research clearly indicates that the burden of organizing and coordinating the care for individuals with PWS rests on the caregivers themselves. Finally, the socio-cultural determinants of the presented way of experiencing PWS by caregivers of children with this syndrome include low public awareness of this issue, as well as limited knowledge of some doctors about PWS, its symptoms, course and therapeutic options.

### Limitations of the study

The analysed group of PWS caregivers may not be representative of the entire community of parents looking after PWS individuals as the consent to take part in the study could have been predominantly given by the most empowered parents displaying a proactive approach to coping with their children’s problems (referred to as “*sharks*” by Gómez-Zúñiga et al. [1]). It seems that in our research, we may have been faced with a particular social population providing largely effective care for the PWS individuals (in the vast majority of cases). Parents with such qualities may be overrepresented in the analysed population, hence the illness experience described on the basis of their interviews may be most applicable to proactive parents. Our results may not sufficiently reflect the experience of passive parents (referred to as “*happy flowers”* by Gómez-Zúñiga et al. [1]). We cannot be certain whether the specificity of the illness experience of passive parents, less effective or entirely ineffective in terms of care provided to their children, would be analogous. One should also remain cautious when generalizing the results given the significant predominance of women in the analysed group of caregivers of PWS individuals.

Moreover, we do not claim that all caregivers of individulas with PWS must necessarily go through all the stages of experiencing PWS described in our research (although the response to the diagnosis, the same or different, seems to be a common element opening the process of experiencing the illness), or experiencing PWS in the manner described in our article. We agree with the thesis of Charles A. Corr [47: p. 53], who, when discussing the concept of E. Kűbler-Ross, pointed out that “a good model for coping with dying should emphasize both that which is universal and that which is individual”. This remark also applies to our research area: the created generalization is intended to help understand the problems of caregivers of individuals struggling with PWS, while leaving space for individual and contextual differences in this regard. We are aware that the process of experiencing PWS described in the article concerns a specific social group, studied at a specific time, place and social context; therefore, other caregivers of individuals with PWS may experience the illness in a different way. The presented findings concern only the period of experiencing PWS by caregivers of persons with this condition, which was accessible and was the object of study.

### Data reporting

Reporting of qualitative research was carried out in accordance with the criteria of the COREQ checklist [48].

## Conclusions

- The illness experience of caregivers looking after individuals with PWS is approached in terms of a response to a situational crisis resulting from manifestations of the specific, behavioural PWS phenotype.
- The study facilitated the identification of phases of the illness experience process from the perspective of the caregivers of individuals with PWS. The results of our study indicate that the support provided to families looking after PWS individuals ought to take into account the processual as well as the gradual character of the course of the illness, which will facilitate adapting the support to the evolving problems and needs of PWS families.

### Practical implications of the results

As observed by Strauss and Corbin [35: p. 40–43], the patients themselves and their family members should constitute the primary source of data regarding the illness experience. The authors posit that learning about the patient’s perspective is a condition for optimising care provided in the course of chronic illness, in which curing the patient is not possible and the primary goal is to provide care and support for families looking after individuals with the illness.

Describing the specificity of the illness experience from the perspective of the individuals with PWS and their families will serve to increase the awareness of medical practitioners that living with a chronic disease is not limited solely to its biological aspects but also implies processes taking place in the “patients themselves” as well as their caregivers in terms of their personal and social identity, biographical issues, etc. [35: p. 279]. Our study was inspired by the belief that designing effective activities to support PWS families ought to begin by actively listening in order to better understand their specific needs.

## Supporting information

S1 File In-depth interview. (DOCX)

S2 File Consent of bioethics committee. (PDF)
