# Supplementary material for: Experiencing illness as a crisis by the caregivers of individuals with Prader-Willi Syndrome
Source: PLoS One. 2022 Sep 1;17(9):e0273295. doi: 10.1371/journal.pone.0273295 (PMC9436047; doi:10.1371/journal.pone.0273295)
Supplement: S1 File — (DOCX) [file pone.0273295.s001.docx]

Katarzyna Kowal DHum.

Faculty of Health Sciences

Jan Długosz University of Humanities

and Life Sciences in Czestochowa

***Dear Sir/Madam,***

*My name is Katarzyna Kowal, I am a medical sociologist conducting sociological research on the issue of experiencing Prader-Willi syndrome by caregivers of individuals with this rare genetic illness. I am interested in the ways in which a family deals with problems resulting from a child's illness, taking into account the impact of the illness on social interactions within the family and further social surroundings, as well as the meanings assigned to the illness.*

*I hereby kindly request you to participate in a sociological study, which will take the form of an in-depth interview. Participation in the study is voluntary. You have the option to resign from participation in the study without giving a reason or to terminate the interview at any time during its duration. As the person carrying out this study, I ask you to take all the questions seriously as well as to provide honest and complete answers. Your participation in the study is anonymous and all the information obtained therein will be used solely for research purposes. I would also like to assure you of the legal protection of the materials collected during the study, which will not be disclosed to anyone. Their publication will only take the form of quoting excerpts of statements that, in accordance with the principle of confidentiality, will not allow you to be identified as a study participant.*

*The administrator of the data collected during the interview is Jan Dlugosz University in Czestochowa - in accordance with the Regulation on the Protection of Personal Data (GDPR) of the European Union.*

*Thank you very much for agreeing to participate in the study.*

**In-depth interview with a caregiver of an individual with PWS**

*At the beginning, the researcher asked for information about individual with PWS: sex, age and the age at which the diagnosis of PWS was made.*

**DIAGNOSIS OF PWS IN THE EXPERIENCES OF CAREGIVERS**

1. What was your reaction to the diagnosis of Prader-Willi syndrome in your child?
2. How did the diagnosis change the life of your family?
3. How is your family's life different from that of other families?
4. To what extent has the illness disrupted your family's life? In which areas of everyday life are these disturbances felt most strongly?

**THE WORK OF THE FAMILY ON THE ILLNESS**

1. How do you as a family work on this illness?
2. How many people in your family participate in the work on this illness? Can you speak of teamwork here? If so, please describe it.
3. Do you as a family have any organization of work on this illness? If so, what organizational strategies have you developed?
4. Do you as a family have any arrangements for working on this illness - for example, who, how, where and at what time will they perform this work? If so, what do these arrangements look like? Between whom are they made?
5. Who is the more active party between the parents in managing this illness?
6. How do other family members – e.g. siblings of the child with PWS cope with this illness?
7. Do these actions that you take as a family influence the course of this illness? If so, how is it manifested?

**THE IMPACT OF THE ILLNESS ON THE FAMILY**

1. How does the illness affect your family?
2. Is there a perceptible positive impact?
3. Do you see any value or sense of this illness and that your child is ill with it?
4. Can you talk about any "benefits" of this illness or its positive sides? If so, are there ones you have experienced personally?
5. What are the interactions in your family in the context of the illness? How do you describe them? Has the illness changed them somehow? If so, what do these changes depend on?
6. Do you talk about the illness in the family? If so, how do they go? Does the child with PWS participate in these conversations?
7. How does the illness affect your socio-economic position as a family?

**SYMPTOMS AND EFFECTS OF THE ILLNESS IN THE EXPERIENCE OF CAREGIVERS**

1. Who in your family, in addition to the child with PWS, is affected by the effects of this illness?
2. Which of the symptoms of the child's illness do you feel is the worst? Why this symptom exactly?
3. Was there something unexpected for you in the child's illness? If so, what did this element of surprise consist in?
4. How do you deal with the consequences of your child's illness?
5. What do you do to deal with the child's illness as a caregiver?
6. How does your child experience the illness? How do you react to this way of your child experiencing the illness?
7. What significance do you give to the fact that your child is ill with it? When experiencing this illness, did you ask: "Why did this happen to my child?" If so, did you manage to find the answer to this question?
8. What is the significance of this illness for you? Have there been any changes in your person as a result of the illness? If so, what changes do you see?

**SOCIAL SUPPORT**

1. Does anyone, and if so, who supports you in dealing with your child's illness? What are the forms of this support?
2. Is the received support sufficient? Please justify your answer.
3. At what point in experiencing the child's illness did you need the most support? Why were these needs the greatest at this point?
4. What kind of support do you expect in the context of the child's future?

**THE FUTURE OF AN INDIVIDUAL WITH PWS IN THE PERSPECTIVE OF A CAREGIVER**

1. Do you think about the future of your child?
2. Are you planning it? If so, please try to formulate these plans.
3. What are your hopes, fears or anxieties regarding your child's future?
4. Do you see it optimistically or pessimistically?
5. What is the best scenario for the future that you see for your child?

**DEMOGRAPHIC QUESTIONS**

1. **Sex**
   1. female
2. male
   1. **Age** (please give your age on your last birthday)......................years old
3. **What is your relationship to the individual with PWS?**..............................................
4. **What is your marital status?**
   1. single
      1. married
      2. divorced
      3. widowed
      4. other (which?)...........................................................................................
5. **What is your level of education?**
   1. elementary
      1. vocational
6. secondary
7. post-secondary
8. **Your permanent place of residence:**
   1. countryside/village
   2. town to 50,000 inhabitants
   3. city from 50,000 to 100,000 inhabitants
   4. city over 100,000 inhabitants
9. **What is your current professional situation?**
10. I run a farm
11. I work in a state-owned (local government) company
12. I work in a private company
13. I run my own business
14. I do not work professionally, I am unemployed
15. I do not work professionally, I am a pensioner
16. other answer (which?).......................................................................................
17. **Are you:**
    1. a practicing believer
    2. a non-practicing believer
    3. a non-practicing non-believer (please skip question 10)
    4. practicing non-believer (please skip question 10)
    5. other answer (which?).......................................................................................
18. **What is your religion?**................................................................................
