# Supplementary material for: Experiencing illness as a crisis by the caregivers of individuals with Prader-Willi Syndrome
Source: PLoS One. 2022 Sep 1;17(9):e0273295. doi: 10.1371/journal.pone.0273295 (PMC9436047; doi:10.1371/journal.pone.0273295)
Supplement: S2 File — (PDF) [file pone.0273295.s002.pdf]

KOMISJA BIOETYCZNA
przy Uniwersytecie Medycznym w Lublinie
20-059 Lublin, Al. Racławickie 1
tel. 81 448 52 13, fax 81 448 52 11

UCHWAŁA KOMISJI BIOETYCZNEJ

numer KE-0254/253/2019

W dniu **27 czerwca 2019 r.** Komisja Bioetyczna przy Uniwersytecie Medycznym w Lublinie, Al. Racławickie 1 zapoznała się z projektem badania:

„Doświadczenie zespołu Pradera – Williego z perspektywy opiekunów chorego dziecka – socjomedyczne studium rzadkiej choroby genetycznej"

Projekt przedstawia:

dr hab. Michał Skrzypek
Zakład Dietetyki Klinicznej
Uniwersytet Medyczny w Lublinie

Do Komisji wpłynęły następujące dokumenty:

Protokół badania
Informacja dla badanego
Formularz zgody badanego na udział w badaniu
Wytyczne do wywiadu z opiekunem uczestnika badania

Po zapoznaniu się z całością dokumentacji, zgodnie z zasadami GCP (Guidelines for Good Clinical Practice), Komisja Bioetyczna:

wyraziła **pozytywną** opinię o przedstawionym projekcie badania.

PRZEWODNICZĄCY
Komisji Bioetycznej
dr hab. n. med. Marcin Olajossy

.... Przewodniczący Komisji

2019 06 27
.... data

Członkowie Komisji Bioetycznej:

KOMISJA BIOETYCZNA
Uniwersytecie Medycznym w Lublinie
-059 Lublin, Al. Racławickie 1
81 448 52 13, fax 81 448 52 11

LISTA CZŁONKÓW KOMISJI BIOETYCZNEJ
opiniujących Uchwałę KE-0254/253/2019
z dnia 27 czerwca 2019 roku

Lp.	Imię i nazwisko	Zawód	Podpis
1.	dr hab. Marcin Olajossy	psychiatra	[signature]
2	ks. prof. dr hab.Andrzej Szostek	duchowny	
3.	prof. dr hab. Jolanta Szymańska	stomatolog	[signature]
4	prof. dr hab. Paweł Krawczyk	internista	
5	prof. dr hab. Andrzej Dąbrowski	chirurg ogólny	[signature]
6	prof. dr hab. Wojciech Polkowski	chirurg onkologiczny	
7	dr hab. Elżbieta Czekajska – Chehab – prof. nadzw.	radiodiagnosta	
8	dr hab. Iwona Beń - Skowronek	pediatra	[signature]
9	prof. dr hab. Janusz Kocki	genetyk	
10	dr hab. Michał Skrzypek	internista, socjolog	
11	dr Hanna Czekajska - Łuckiewicz	farmaceuta	[signature]
12	dr Lidia Sierpińska	pielęgniarka	[signature]
13	mgr Stanisław Pęzioł	radca prawny	
